# *Measurement for Change*: Reflections from innovators' experiences with monitoring, evaluation, and learning systems for Early Childhood Development

**DOI:** 10.3389/fpubh.2023.1021790

**Published:** 2023-03-16

**Authors:** Joost de Laat, James Radner, Penny Holding, Lotte van der Haar, Wiedaad Slemming, Joachim Krapels, Maria van der Harst, Abbie Raikes, Anselme Simeon Sanou, Caroline Dusabe

**Affiliations:** ^1^Utrecht University Centre for Global Challenges, Utrecht University, Utrecht, Netherlands; ^2^Munk School of Global Affairs & Public Policy, University of Toronto, Toronto, ON, Canada; ^3^Identitéa/Affiliate of Utrecht University Centre for Global Challenges, London, United Kingdom; ^4^Department of Paediatrics and Child Health, School of Clinical Medicine, University of Witwatersrand, Johannesburg, South Africa; ^5^Porticus, Amsterdam, Netherlands; ^6^College of Public Health, University of Nebraska Medical Centre, Omaha, NE, United States; ^7^Public Health Department, Centre Muraz Institute, Bobo-Dioulasso, Burkina Faso; ^8^Save the Children Australia, Melbourne, VIC, Australia

**Keywords:** monitoring, evaluation, learning, Early Childhood Development, scaling, *Measurement for Change*

## Abstract

In this review paper, we explore how on-the-ground Early Childhood Development (ECD) innovators are using monitoring, evaluation, and learning (MEL) systems to guide the design and implementation of ECD programs, as well as how MEL systems can influence policy and support the achievement of impact at scale. We reflect on articles in the *Frontiers* series “*Effective delivery of integrated interventions in early childhood: innovations in evidence use, monitoring, evaluation, and learning*.” The 31 contributions to the series reflect the breadth and depth of complexity that characterizes ECD, including global geographic spread, with studies from Asia, Europe, Africa, and Latin America and the Caribbean. Our synthesis finds that integrating MEL processes and systems into the fabric of a program or policy initiative can broaden the underlying value proposition. Specifically, ECD organizations sought to design their MEL systems to ensure programs fit the values, goals, experiences and conceptual frameworks of diverse stakeholders, so that participating makes sense to all. For example, formative, exploratory research identified the priorities and needs of the target population and frontline service providers, and informed the content and delivery of an intervention. ECD organizations also designed their MEL systems to support a shift of accountability toward broader ownership: They included delivery agents and program participants alike as *subjects* rather than *objects*, through active participation in data collection, and by providing opportunities for equitable discussion of results and decision-making. Programs collected data to respond to specialized characteristics, priorities and needs, embedding program activities into existing day-to-day routines. Further, papers pointed to the importance of intentionally involving a variety of stakeholders in national and international dialogues to ensure that diverse ECD data collection efforts are aligned and multiple perspectives are considered in the development of national ECD policies. And, several papers illustrate the value of creative methods and measurement tools to integrate MEL into a program or policy initiative. Finally, our synthesis concludes that these findings align with the five aspirations that were formulated as part of the *Measurement for Change* dialogue, which motivated the launch of the series.

## 1. Introduction

Recent influential series on Early Childhood Development (ECD), in 2016, 2018, and 2019,^1^ highlight the important contribution of monitoring, evaluation and learning (MEL) systems in guiding the implementation and scaling up of effective ECD programs. Against this background, *Frontiers in Public Health* launched the series: “*Effective delivery of integrated interventions in early childhood: innovations in evidence use, monitoring, evaluation, and learning*.” This provided a forum for ECD implementers to explore the practical solutions they are generating as they integrate MEL systems into the cycle of program design, redesign, implementation, adaptation, and scaling. Altogether, 31 articles were accepted for publication.

The *Frontiers* series built on a year-long (2019–2020) dialogue among ECD practitioners, researchers and MEL specialists working in low-resource settings across the world. The outcome of this dialogue is described in detail in Krapels et al. (1), while van der Haar et al. (2) describe the consultative process involved. The dialogue, facilitated by authors of this paper, was built around the concept of *Measurement for Change*. The goal was to explore the contribution MEL systems can make in both capturing and supporting meaningful change, both at the ECD program level and in national and international policy. The dialogue considered how MEL can become more than a program “add-on” by integrating into collaborative decision making for effective delivery, reaching beyond those tasked with running MEL systems.

With these ambitions in mind, participants in the dialogue articulated a set of *aspirations* for MEL systems that support meaningful and durable improvements for ECD program beneficiaries (1). These aspirations, which together animate the *Measurement for Change* approach, are for MEL systems to be:

**Dynamic**: With the capacity to adjust frameworks, processes, or methods to be responsive to challenges, surprises, or opportunities, and to be able to reach learning goals.
**Inclusive**: With the capacity to identify and actively involve all stakeholders in making contributions to, and benefiting from, measurement and learning.
**Informative**: With the capacity to continuously seek, assess and use information from various sources to guide decision-making.
**Interactive**: With the capacity to observe, track and utilize interactions, responses, and relationships.
**People-centered**: With the capacity to be responsive to distinct and different goals, strengths, priorities, circumstances, characteristics of different people and communities” [(1), p. 3].

The majority of the articles in the recent *Frontiers* series were written by participants in the *Measurement for Change* dialogue, sometimes in co-authorship or consultation with authors of this review paper (2). This paper is not intended to summarize all the articles in this series, nor even to touch on all the major insights. Instead, by drawing on selected experiences reported in the series, we explore how on-the-ground ECD innovators are using MEL systems to guide decision-making, and ask to what extent the aspirations of *Measurement for Change* are evident in their narratives.

## 2. Overview of the publications

The geographic spread of the 31 contributions in the *Frontiers* series includes Asia (India, Pakistan, Bangladesh, Tajikistan, China), Europe (Poland, Austria, Romania, Bulgaria), Africa (Cameroon, Malawi, Kenya, South Africa, sub-Saharan multicountry), and Latin America and the Caribbean (Peru, Brazil, Colombia, Jamaica), with some countries hosting multiple studies (e.g., India). In terms of ECD intervention areas, some papers focused primarily on health, with programs addressing, for example, anemia, nutrition, oral hygiene, and a public mHealth system. Two papers were concerned with assessment instruments for child development in general, and language in particular. Other papers featured parenting programs as the main, or an important, intervention focus. A major theme across papers was the value placed upon community engagement. With families and education being at the center of these narratives, the age of the children involved ranged from the prenatal period through to the age of six, the full ECD period.

Our reflections begin with a group of papers that illustrate how MEL supported program design and implementation, creation of novel approaches, or adaptation to new contexts. We then discuss papers that describe specific MEL techniques or methods, which the developers have also used to support a cycle of review and reflection. The third group of papers report on the use of existing implementation science frameworks to guide the implementation process and its evaluation. We next consider papers that report experiences using MEL systems and data more broadly, to support scaling efforts and policy change. Finally, we discuss papers that describe strategies to consolidate information, at national or international levels, to guide decision-making in policy and practice.

## 3. Using MEL to guide design, implementation, and fast cycle learning

The papers considered in this section describe how MEL systems collected information to guide the development and delivery of specific interventions, with an eye to process, quality and in some cases impact. The results support the longer-term goal of building a delivery system for an intervention to achieve sustainable impact at scale.

Krishna et al. (3) uses two case studies to describe how the NGO Amar Seva Sangam (ASSA) used MEL to improve the design and implementation of an early childhood intervention in India. The program's objectives included early identification of children with delayed development, and effective support for their caregivers through early intervention services. ASSA delivered services to families through in-person visits and a mobile technology platform. Implementation was informed by a deliberate and detailed cyclical process of data collection, analysis, reflection, and adaptation. One example was the way ASSA addressed challenges faced by female community health workers, who are at the heart of the home visiting program. ASSA conducted five focus group discussions with 25 female staff, guided by issues raised in publications from other settings in India (4), as well as those captured in the Gender Equality Strategy Tool developed by Grand Challenges Canada (5). The fears and concerns shared by the female workforce included lack of access to bathrooms and prevalence of menstrual stigma; these in turn were leading to missed workdays. In response, ASSA identified safe bathroom and rest spaces and adjusted the schedule of home visits to enable female staff to access facilities at appropriate intervals. In another example, the paper describes the use of qualitative research and a longitudinal cohort study to explore drivers of low program engagement and low school attendance among children with delayed development. ASSA identified lack of caregiver peer support as one such driver and created parent participation groups to address it.

Nair et al. (6) describe how innovators in Tamil Nadu, south India collected quantitative and qualitative data to inform the design and implementation of a program to promote fathers' involvement in parenting. The program worked within the Integrated Child Development Services scheme (ICDS), a flagship ECD initiative of the Government of India. The initial design was informed by key informant interviews conducted with fathers, mothers, Anganwadi Workers (the delivery agents), district ICDS teams, and statewide leaders. The team also analyzed government datasets for additional contextual information. To investigate impact, the team conducted a cluster randomized trial (reported separately) and a series of qualitative longitudinal case studies. To refine the model in preparation for scaling, the team analyzed information on beneficiary engagement, drawing on the longitudinal study and cross-referencing data from the impact analysis. The program also engaged with *mothers* from the beginning, and the MEL system tracked related dynamics. This serves as an example of M*easurement for Change*, as the authors observe that “*[t]hough mothers initially served as barriers and gatekeepers in the way of father-child bonding, they eventually became enablers in the process*” [(6), p. 12].

Apte et al. (7) describe how a team from the Indian Institute of Technology Bombay (IITB) embedded MEL in a Theory of Change (ToC) approach to create an intervention to deliver essential micronutrients to infants. The intervention builds on an “*ancient cultural practice of gentle oil massage for infants*” [(7), p. 1]. Working collaboratively with scientists and community members, the team used a series of sub-studies, of varying sizes and duration, some quantitative and others qualitative, to assess technical, social and cultural considerations that would be critical for success. For example, an initial questionnaire among 201 households revealed that massage commonly occurs just before infants were bathed, and identified a potential risk to dosage levels, since oils would be washed off. Based on this information, the team developed guidelines for an acceptable bedtime massage routine, for optimal transdermal uptake of micronutrients. The team also assessed the efficacy of its pilot through a clinical trial. The authors conclude that collaborative, systematic evaluation of causal pathways can effectively guide decision-making and adaptation on the path to scale.

Dzabala et al. (8) describe a year-long MEL process to build an evidence base for the design of a program to support young mothers and their children in three districts in Malawi. A collaboration between the Young Women's Christian Association (YWCA) and the University of Malawi, the team combined evidence from scientific and gray literature, lessons learned from prior projects with a similar focus in the local context, and a baseline survey among 135 adolescent mothers to test assumptions about the priorities and needs of end users, and to guide adjustments to the project design. For example, one a priori assumption was that children born to adolescent mothers would have lower birth weights. Baseline data revealed that this assumption was incorrect: the challenge that needed to be addressed was not growth, but rather achievement of developmental milestones in a wide range of domains, e.g., socio-emotional, cognitive, and motor development. The team therefore expanded the intervention to cover those domains, with attention to maternal wellbeing as a critical component. The evidence that young mothers were struggling with stress and low self-esteem motivated the creation of peer support groups [analogous to those described in another paper in the series, Kachingwe et al. (9)].

Similarly, Muthuuri et al. (10) describe how “A Mile for the Brain” used formative research to inform the design of a program to address malnutrition in children aged 6–24 months, in two counties in Kenya. The program engaged local women as entrepreneurs to increase the availability and uptake of commercially available, high-protein complementary foods. The formative research involved an iterative process of consultations with mothers and women entrepreneurs, as well as other stakeholders such as staff with the Ministry of Health. The team used the findings to selected foods that children favored, and that could be locally sourced. Further, research identified a challenge to achieving required hygiene standards in home-based repackaging. In response, the team successfully negotiated with a food manufacturer to create small food sachets at an affordable price. Consultation was key for this program to generate the information needed to increase benefits and reduce risks.

The narratives reviewed above illustrate the value of building feedback loops into a MEL system, where various stakeholders such as community members and program staff have voice and genuine engagement, which enables the implementation to respond rapidly to challenges. The example below illustrates how this inclusive approach can lead to beneficial effects in domains not intentionally examined by the MEL system.

González-Fernández et al. (11) report on a multi-sectoral approach to improve health and developmental outcomes for children from birth to age three living in peripheral settlements in Lima, Peru. The program consisted of home gardens, nutrition workshops, and workshops focused on caregiver-child interactions. The MEL system tracked and evaluated multiple outcomes, including child development, feeding, food security, and caregiver behaviors. It also included weekly meetings with the frontline health workers, called “*community health promoters”* [(11), p. 1]. Change was explored through analysis of both quantitative and qualitative measures tracking the pathway from the training of mothers as community health promoters, through their inclusion in the adaptation and delivery of the program, to the achievement of positive child outcomes. The use of open and inclusive dialogue led to a beneficial, yet unintended effect: “*the empowerment, enhanced communication skills and increase in self-confidence*” [(11). p. 15] of community health promoters.

Changes in the status of service providers, whether intentional or unintentional, were also reported by Muthuuri et al. (10) and Krishna et al. (3). These results suggest that empowerment, confidence building, and enhanced status for these stakeholders is not only inherently valuable, but also potentially an important pathway for the effective delivery and the durability of interventions. In the following two projects, MEL supported the introduction of additional responsibilities for people serving families.

Mehrin et al. (12) describe the role of MEL as the International Centre for Diarrhoeal Disease Research, Bangladesh (Icddr,b) and partners prepared the Reach-Up and Learn curriculum for implementation within the government community health services in rural Bangladesh. Reach-Up and Learn (13) is an international program, developed by researchers at the University of the West Indies, to promote caregiver-child interactions and learning for young children through a series of home visits. Icddr,b had previously shown, in two randomized controlled trials, that versions of the curriculum were effective in Bangladesh. The challenge addressed in this paper was to integrate the program into the government community health system. A key consideration was the workload demands being made on community health workers, the intended delivery agents. The team adapted the original curriculum, notably for delivery in clinics rather than home settings; piloted two alternative group delivery models; and tracked both implementation processes and child outcomes. Focus group interviews with mothers and local health workers, alongside monitoring of attendance at meetings with families, identified a series of areas for further adaptation. For example, the MEL system uncovered that continued participation by mothers was more difficult in a clinic setting than at home. To promote participation, the team introduced motivational community meetings, calibrated the number of sessions per week to fit workloads, and made session content more simple and collaborative. The authors recommend continued, iterative rounds of implementation, evidence collection and participant feedback as an overall strategy for adaptation to new contexts.

Slemming et al. (14) describe the use of MEL in the Healthy Pregnancy, Healthy Baby (HPHB) program, a hospital-based intervention that integrates promotion of nurturing childcare into routine maternal antenatal care. The program, in Soweto, South Africa, used ultrasound sessions to share with parents information on fetal and infant development and on the importance of parent wellbeing for infant care. The team established a broad stakeholder network from the beginning, and used formative research involving interviews with pregnant women to inform program design. These methods “*confirmed the importance of including partners [generally fathers] in the intervention and the need for information on their role during pregnancy and parenting*” [(14), p. 5]. A workload issue here emerged again as key, as the research highlighted that the intervention needed to be “*light touch*” [(14), p. 5], for feasible delivery by sonographers. During implementation, the team developed a dashboard to monitor key indicators, and they organized regular “*whole team*” [(14), p. 7] meetings to discuss data collection and review findings. This enabled timely, informed adjustments to the program, including revision of the dashboard itself. The paper emphasizes the value of creating “*dynamic, inclusive and interactive approaches to intervention development and implementation*” [(14), p. 1].

Shaw and de Cácia Oenning da Silva (15) document a case study from the CanalCanoa program where indigenous communities took center stage in the development of program content, program delivery, and MEL. CanalCanoa works in Brazil with indigenous communities in the Amazon Basin under pressure from urbanization and colonization. The intervention intentionally facilitated opportunities for indigenous communities to reflect on and adapt traditional practices and knowledge in the promotion of early child development. Through videography, including short films made by children themselves, indigenous communities created a bank of materials documenting traditional child-rearing practices and perspectives. These materials served as core content to trigger reflective discussion in community meetings, led by community members. MEL work was carried out through interviews with participants in the meetings, with the results fed back immediately into program design. For example, early on (and consistently thereafter) participants noted the connection between reflections on early childhood practices in the community meetings and the resulting opening up of space for interactions within and across communities that urbanization had seemingly blocked. This finding encouraged continued investment in the community sessions and emphasis on social connections as the program proceeded. In sum, the program as a whole functioned as a collaborative learning system. Shaw and da Silva report, “*In the language developed by the Measurement for Change initiative, the entire intervention was* inclusive*: the community contributed to the development of the project, benefited directly from learnings, decided on indicators, provided data, and in the end had access to all elements of the data and conclusions*” [(15), p. 4, emphasis original].

## 4. Tools and methods that combine measurement of and for change

Reflective discussions with the community around materials and processes also played a key role in the program delivery and development described in three papers considered below, which reported on specific measurement tools and methods.

Gaidhane et al. (16) describe how photographic records (“photostories”) provided the Stepping Stones program in rural communities in central India with a rich source of information about local parenting practices that guided training for home visitors on a parenting curriculum. Sharing photostories in group sessions with caregivers and other community members triggered reflection and discussion, serving as a distinctive, effective basis for community engagement. The photostories were used to both monitor change in child care practices and engage the community in generating further change. A standardized rating system illustrated existing positive practices, e.g., the way parents provided children with opportunities to play and explore, as well as targets for change, e.g., the appropriate matching of learning activities to children's developmental level. The ratings also highlighted changes in parental behaviors following the intervention. For example, at baseline, “*the photostory would describe young children sitting alongside a busy parent. At endpoint, the photostories showed parents involving their children directly in the activity, increasing both the engagement of the child, and the level of communication between parent and child. This level of detail, embedded as it is in the local setting, provides data that is accessible and meaningful to the community*” [(16), p. 9].

Muhamedjonova et al. (17) discuss integration of a MEL network mapping tool into a program in Tajikistan designed to shift the delivery of support for vulnerable children from institutional care, in publicly run Baby Homes, to family-based care, supported by Family and Child Support Centres. Mapping networks available to caregivers helped the team to monitor the way caregivers sought and used available support. Service providers and caregivers collaborated to develop and reflect on the maps, enabling learning at multiple levels. A review of the data identified the need for new program components, for example: group-based support for mothers with anxiety and depression to help build positive relationships; and a suite of activities for fathers, who were commonly unaware of available support services. The mapping also revealed that the main government agency operating at the neighborhood level had yet to be included directly in the implementation process. At the program level, the team gained a greater understanding of how their intervention's theory of change unfolded in practice, and program staff gained awareness of the value of systematic and regular review of practices. At a more general level the results both “*brought to life*” and “*add[ed] an extra dimension*” [(17), p. 8] to Bronfenbrenner's Ecological Systems Theory (EST) (18), by identifying the need to structure support in a more networked rather than a nested distribution. The authors quote Neal and Neal (19) in this regard, pointing to an insight potentially relevant to policy design in similar contexts. The value of the network mapping process extended beyond the data collected by the tool, to include social benefit to both individual participants and the program as a whole, arising from the opportunity to meet, to make connections and to enhance participant voice, thus further strengthening individual support networks.

Anziom et al. (20) take community engagement in measurement one step earlier in the MEL process, by working together with the community to create a valid and reliable measure of change. This paper highlights how building capacity to measure change can begin with indigenous concepts and constructs and use participatory methods and local expertise to develop corresponding instruments and procedures, while also drawing upon international expertise. The study describes the development of the Socio-Emotional Learning (SEL) framework and measurement tool among the indigenous Baka communities in Cameroon. The team recognized that while tools developed outside this community could inform a conceptual understanding of SEL, attempting to adapt those tools for local use “*risks carrying over assumptions about valuable skills from one culture to another*” [(20), p. 2]. Therefore, the team developed the new SEL framework “*from the ground up with caregivers*” [(20), p. 3], starting with indigenous concepts and perspectives. The team established key constructs that defined successful social and emotional development, through multiple stages, using local narratives and question- and-answer sessions. The authors observed that “*the Baka SEL framework displays high regard for skills and behaviors that support collaboration and community wellbeing, and notably lacks those related to individual identity and goals”* [(20), p. 15]. The local community continued to play a key role in item selection and definition, and in guiding the design of administration procedures. At the same time, the team sought confirmatory evidence of the reliability of the tool, and of its contribution to a more global understanding of SEL. In this phase of the study, the team collected and analyzed more quantitative information.

## 5. Applying standard evaluation frameworks

Three papers reviewed here discuss how the use of standard evaluation frameworks guided systematic collection of inputs from a wide range of stakeholders to inform decisions across each program's lifecycle.

Westgard and Fleming (21) detail the selection and use of the *Active Implementation Framework (AIF)* (22) in the design and implementation of a community-based mHealth intervention called the Child Health Education and Surveillance Tool Application (the CHEST App). Community health agents used CHEST in managing child health in the Amazon region of Peru. AIF distinguishes four stages involving continuous, iterative cycles of learning and modifying, from (1) “exploration” through (2) “installation,” (3) “initial implementation,” and (4) “full implementation.” For example, an Acceptability Assessment Form allowed the team to identify ways to improve the CHEST App, including: using more localized terminology to increase message clarity; tighter control on the selection of images, to avoid confusion; and flexibility in the recording of a child's ID number, to account for children without government-issued IDs. These adaptations helped the program achieve high rates of fidelity and acceptability in its distinctive community setting. As the authors conclude, “*The AIF highlighted several potential barriers to implementation that may have been overlooked without the guidance of a science-based implementation tool”* [(21), p. 1].

Francis and Baker-Henningham (23) selected a different evaluative framework, the *UK Medical Research Council Guidance on Developing and Evaluating Complex Interventions* (24), for their work to develop the Irie Homes Toolbox in Jamaica. The toolbox is a violence prevention program for use with parents of children aged 2–6 years. The evaluative framework, like the AIF described in Westgard and Fleming (21), included four stages. Stage one, preliminary design, included efforts to learn from other evidence-based parenting programs, matching to the perspectives of the end users, Jamaican parents. This was followed by further formative research around a small, preliminary pilot with iterative adjustments (stage 2) to yield an “initial draft” intervention design. This was in turn modified through a second small pilot (stage 3). The “final draft” of the intervention design was assessed through a small-scale efficacy trial and a process evaluation (stage 4). The net result, achieved over a period of 15 months, was a project design ready for large-scale trials. The authors highlight how design decisions were informed by “*an approach that integrates formative research, theory, empirically derived content and behavior change principles, with extensive piloting in the context*” [(23), p. 17], to assure “*the acceptability, feasibility, relevance, and effectiveness of the intervention with the target population*” [(23), p. 17].

Finally, Luoto et al. (25) report on the use of the Consolidated Advice for Reporting ECD implementation research (CARE) guidelines (26) by the Msingi Bora (“Good Foundation” in Swahili) program, a responsive parenting and nutrition education program in Western Kenya. As the authors note, the CARE guidelines provide a structured framework for understanding and reporting implementation in a way that addresses previously existing gaps in relevant literature, for example by assuring attention to “*contextual factors...such as how the needs of parents influenced the program content, and how the capacity of providers influenced the training offered*” [(25), p. 2], as well as “*the determinants and consequences of implementation outputs*” [(25), p. 2]. Before the full implementation, Msingi Bora conducted a small pilot, where the team identified, for example, a “*need to reduce lengthy speeches by the CHV [community health volunteers] in favor of practical activities*” [(25), p. 4]. Examining the full implementation *via* the CARE framework and related published measurement strategies (27), Msingi Bora systematically captured and evaluated key implementation linkages, from inputs to outputs and outcomes. For example, the paper reports on details of preparation and training, the quality and fidelity of delivery, and participant engagement. A complementary RCT effectiveness evaluation enabled the team to connect delivery fidelity to parent and child outcomes. A key finding was the importance of “*upfront investment in training local trainers and delivery agents, and regular supervision of delivery of a manualized program*” [(25), p. 15]. In sum, the authors provide detailed documentation of the dynamics of a successful implementation of an effective parenting intervention and conclude that the “*results represent a promising avenue for scaling similar interventions in low-resource rural settings to serve families in need of ECD programming*” [(25), p. 15].

## 6. MEL in support of scaling up interventions and changing public policy

The three papers described below discuss the role of MEL in the process of scaling up interventions developed by civil society organizations, where the scaling strategy involved public sector adoption, collaboration, or support. In each case, scaling required the MEL approach to be adapt over time, engaging diverse and sometimes changing sets of stakeholders, from end beneficiaries to local and national government authorities. This process spanned the life cycle of the program, from initial buy-in and project design to large-scale implementation or adoption by public sector authorities.

The paper by Mesa et al. (28) illustrates how different MEL activities helped the scaling of “aeioTU,” a center-based early childhood program in Colombia providing children with high quality ECD services inspired by the Reggio Emilia approach. As the authors report, “*In January 2009, aeioTU opened its first for-profit center in Bogotá and two fully subsidized centers in low-income communities in Barranquilla and Bogotá*” [(28), p. 3]. By December 2015, aeioTU had 28 centers, funded largely through the public sector and philanthropy. In 2016, it modified its scaling strategy to extend its reach beyond centers operated by aeioTU. The new strategy involved developing support services for existing government early childhood education and development (ECED) centers and expanding reach through a new internet resource platform, a professional development and capacity building program, and a membership network. As the authors note, “*As of December 2019, aeioTU had reached 228,667 children in 1,851 ECED centers*” [(28), p. 6]. The paper emphasizes that evaluation and continuous learning played a key role in aeioTU's adaptation and scaling process. For example, aeioTU used the Balanced Scorecard technique to continuously assess progress. To demonstrate effectiveness and identify areas for improvement, aeioTU organized a series of process and longitudinal impact evaluations in collaboration with academic institutions. The results, combined with a background of evidence on the importance of ECD, were used to inform and strengthen partnerships essential for scaling, especially with the national government (including at high levels), but also with global partners, for example in philanthropy.

The paper by Gheorghiu et al. (29) also underscores how MEL was essential to generate initial interest and secure stakeholder buy-in for scaling up. The paper describes how a Romanian NGO pre-school project achieved scale through the adoption by the Romanian parliament of a new law. The 2015 law provided food coupons to poor families conditional on children attending preschool. It was modeled on the project “Every Child in Kindergarten,” designed and launched in 2010 by a small local NGO called Asociatia OviduiRo (OvR). The paper highlights how the initial project, like the corresponding work by aeioTU in Colombia, drew on a background of increasing global interest—supported by scientific evidence—in expanding ECD opportunities. Project monitoring was an important part of MEL activities, and the authors note that implementation “*was most successful in communities where the school director, social workers and municipal personnel participated in the monitoring process*” [(29), p. 6]. Findings from a quasi-experimental impact evaluation were “*extremely helpful*” [(29), p. 5] to cement partnerships with local authorities and corporate sponsors, and “*also valuable*” [(29), p. 5] with members of Parliament. After the new law passed, the learning process continued through an evaluation of the implementation of the 2015 law by another NGO. The evaluation led to further recommendations submitted to Parliament and enacted in a 2020 amendment to the law.

Volen and de Laat (30) document a parallel process in Bulgaria, where in 2020 Parliament adopted a law that provides funding to municipalities to remove attendance fees at ECD centers for the poorest 40% of children. The law was modeled on a project, Springboard for School Readiness (SSR), implemented by a non-profit organization, the Trust for Social Achievement (TSA). The 2014 launch of SSR was motivated by international evidence of the importance of investment in early education and was designed to address low kindergarten participation rates among poor children in Bulgaria, Roma children especially. Effectiveness of the SSR project, which included removal of attendance fees, was demonstrated through a World Bank funded RCT conducted in collaboration with academic researchers. However, while the results generated considerable interest, they proved insufficient to motivate key decision-makers to advocate for public policy change. TSA, with the support of a network of local NGOs, built up a portfolio of information to support the advocacy process. They collected both qualitative and quantitative data that documented cases where municipalities had already removed attendance fees, and they combined this information with the RCT's impact estimates. They used a national survey to collect data on citizen attitudes toward policy change and completed a stakeholder mapping of potential influencers. These efforts culminated in the 2020 passage of the new national law. This Bulgarian project, like those in Colombia and Romania, used a collaborative MEL process to bring about change at the national level.

## 7. Consolidating information to guide decision-making

We end this review by reflecting upon three papers that describe initiatives to combine and consolidate data to inform national and international ECD practice, and to support policy change and scaling.

The paper by Mithra et al. (31) illustrates the value of the “*bird's-eye view”* [(31), p. 20] provided by data synthesis across multiple, well-executed systematic reviews (SRs) to guide policy, intervention strategy, and further research. The article synthesized 31 published systematic reviews of interventions to manage anemia in children and adolescents. The authors report that “*[r]esults were favorable for fortification and supplementation with clear reduction in the risk of anemia and increase in hemoglobin levels across all age groups. Other interventions reported by the SRs were inconclusive and suggest further research*” [(31), p. 1]. The paper describes dosing strategies and outcomes for children in different age ranges (infancy through adolescence); reviews potential beneficial effects on anemia and hemoglobin levels, along with possible adverse effects; and identifies gaps in knowledge (e.g., effects on anemia of antimalarial or water, sanitation and hygiene interventions) that suggest potential foci for future research.

The paper by Pushparatnam et al. (32) illustrates possibilities and challenges in achieving a different kind of “*bird's-eye view”* (31), through developing an ECD assessment instrument that captures both universal and local context-specific measurement needs, yet remains short and feasible. The team developed a set of statistical criteria “*to identify items that show robust psychometric properties across countries*” [(32), p. 4], and they undertook a step-by-step analytical process to select items from a database from use of MELQO (an international set of assessment tools) (33) in multiple settings, in the aggregate involving 16,015 caregivers and 24,533 children in 12 countries in Africa, Asia, and Central and South America. The result was a set of 20 items to be assessed through caregiver reports and 84 items for use in direct assessment of children. The authors find that literacy and numeracy items showed relatively consistent performance cross-culturally, while psychosocial items performed less consistently. The authors also note that the core items will often need to be supplemented in particular applications, for example to provide more nuance or to respond to local contexts. The challenge here involves instrument length, given the importance of having a short, feasible scale.

Related, the paper by Raikes et al. (34) illustrates how effective *use* of early childhood data at scale can guide the national early childhood policies. Their study derives lessons from the development and early work of the Consortium for Pre-primary Data and Measurement in Sub-Saharan Africa (CPDMA), a project initiated in 2018 by USAID and the ECD Measure Group at the University of Nebraska Medical Center. The project convened multi-disciplinary and multi-stakeholder teams in four countries—Ethiopia, Liberia, Rwanda, and South Africa—comprising government officials, local university researchers, early childhood education (ECE) practitioners from civil society, and USAID education officers. A Data for Impact framework and toolkit was developed, which guided the country teams to build data-driven ECE systems. The authors highlight the importance of: attending to the composition and perspectives of the teams expected to use these data for decision-making; aligning the scope of the data collected with national ECD priority areas; and covering not only child outcomes, but also processes for achieving and maintaining high-quality program delivery. They suggest that national data dashboards, like the National Early Childhood Development Program's dashboard in Rwanda, could help achieve national alignment.

## 8. Conclusions and recommendations

When Cavallera et al. interviewed implementers of ECD projects in low- and middle-income countries in 2019, their respondents “*agreed that M&E was necessary to guide structured data collection and ensure transparency. Yet interviewees reported that data collection in ECD projects was often seen as intended solely for scientific publications, and of little use for project improvement or addressing implementation challenges*” [(35), p. S47]. These findings were reported as part of the 2019 *Archives of Disease in Childhood* series “Informing design and implementation for early child development programs”. However, the papers in the recent *Frontiers in Public Health* series illustrate that integrating MEL processes and systems into the fabric of a program or policy initiative can create a broader value proposition.

Specifically, the examples discussed above illustrate how ECD organizations sought to design their MEL systems to ensure programs fit the values, goals, experiences and conceptual frameworks of diverse stakeholders, so that participating makes sense to all. This is consistent with insights from behavioral science that interventions are more successful when they create the least possible hassle and fit most naturally with existing habits and behaviors [see also Behavioral Insights Team (36)]. For example, our review of papers in the series highlights how:

A. ECD organizations employed formative research that was exploratory, to identify the priorities and needs of the target population and frontline service providers, and to inform the content and delivery of an intervention. This formative research frequently included program delivery agents.
B. ECD organizations designed their MEL systems to support a shift of accountability toward broader ownership: They included delivery agents and program participants alike as *subjects* rather than *objects* of MEL activities, through active participation in data collection, and they provided opportunities for equitable discussion of results and decision-making.
C. Programs used data in a way that went beyond a focus on averages and responded to specialized characteristics, priorities and needs; this approach supported ECD organizations to embed program activities into existing day-to-day routines, be they work or family routines, for better engagement, impact and durability.
D. Experiences with using ECD data at scale to inform policy point to the importance of intentionally involving a variety of stakeholders in national and international dialogues in a way that respects local diversity and community decision-making. Such involvement can ensure that diverse ECD data collection efforts are aligned and multiple perspectives are considered in the development of national ECD policies.
E. Integrating MEL into the fabric of a program or policy initiative may benefit from creative methods and measurement tools, such as network mapping, photography and film.

The integrated approach to MEL described by contributions to this series aligns with the five aspirations that were formulated as part of the *Measurement for Change* dialogue. The *informative* aspiration underpins this approach in a fundamental sense; papers throughout the series describe the systematic use of data to support decision-making in a cycle of learning. The *inclusive* aspiration expands “learning about” into “learning with” (so that, as noted above, participants are subjects, not objects), and overlaps with the aspiration of being *people-centered* (understanding goals, needs and effects at different levels of granularity). The direct and active inclusion of delivery agents in tracking and monitoring the reaction to, and reception of a program by beneficiaries and delivery agents themselves, and to use this information to make appropriate program adjustments, illustrates how the *interactive* aspiration can build empowerment.

Contributions to the series also illustrate the *dynamic* aspiration—anticipating that information needs will change and that MEL systems must respond accordingly to support each phase of the ECD program, from initial design, to piloting, modifying and further piloting. Dynamic MEL systems respond when new groups of beneficiaries enroll as the program expands, when the mix of delivery agents changes as the program is modified, or when different types of public authorities are drawn in when the program scales up. Similarly, in dynamic MEL systems, a large, formal evaluation like an RCT is not seen as a one-off answer, but rather as part of a series of actions that enable ongoing learning and adaptation on the pathway to large-scale impact. Being *dynamic* therefore involves not only responding to people's priorities and needs, but also selecting appropriate methods to address appropriate questions at appropriate times. Iterative application of measurement, reflective learning, and adaptation thus enables each measure *of* change to become a measure *for* change.

The way the ECD organizations shape these five MEL aspirations in practice creates a strong link to the human rights based approach to development, as advocated by the United Nations (37). ECD implementing teams have tried to design MEL systems that reflect the ethical principle of subsidiarity, which holds that “*decisions about efforts to help others and to attain the common good (e.g., by using knowledge to achieve equity in global health) should, by default, take place at the smallest or most proximate level/scale of organization possible, and only when necessary at a larger or more distant level/scale of organization”* [(38), p. 2]. Abimbola (38) advocates this principle not only on moral grounds, based on equity, justice and human dignity, but also on practical grounds, since local decisions can lead to better adapted programming and to better use of initiative, feedback and learning.

Taken together, the papers in this series show that it is helpful to view the five aspirations of *Measurement for Change* not as a checklist, but as stimulus for reflection in developing MEL systems that truly support the effective delivery of interventions. MEL becomes a constant process of intentional inquiry, nudging program implementers to slow down, reflect, and course correct when necessary, thereby paving the way for meaningful change in the lives of families and communities.

## Author contributions

JL led on writing and editing, but substantial written contributions were provided by PH, JR, LH, WS, JK, MH, AR, AS, and CD. All authors contributed equally to the development of the ideas presented in this review. All authors contributed to the article and approved the submitted version.

## Footnotes

^1^The Lancet series in 2016, “Advancing Early Childhood Development: from Science to Scale”; the 2018 special issue of *Annals of the New York Academy of Sciences*, “Implementation Research and Practice for Early Childhood Development,”; the 2019 *Archives of Disease in Childhood* series “Informing Design and Implementation for Early Child Development Programs.”
